# The Role of NLRP3 and IL-1β in the Pathogenesis of Inflammatory Bowel Disease

**DOI:** 10.3389/fimmu.2018.02566

**Published:** 2018-11-05

**Authors:** Liming Mao, Atsushi Kitani, Warren Strober, Ivan J. Fuss

**Affiliations:** Mucosal Immunity Section, Laboratory of Clinical Immunology and Microbiology, National Institute of Allergy and Infectious Diseases, National Institutes of Health, Bethesda, MD, United States

**Keywords:** interleukin-1β, inflammasome, IBD-inflammatory bowel diseases, NLRP3, IL-18

## Abstract

It is logical to assume that a major pro-inflammatory mechanism, i.e., the NLRP3 inflammasome would play a prominent role in the pathogenesis of the Inflammatory Bowel Disease (IBD) in humans. However, while both studies of murine models of gut disease and patients provide data that the main cytokine product generated by this inflammasome, IL-1β, does in fact contribute to inflammation in IBD, there is no evidence that IL-1β plays a decisive or prominent role in “ordinary” patients with IBD (Crohn's disease). On the other hand, there are several definable point mutations that result in over-active NLRP3 inflammasome activity and in these cases, the gut inflammation is driven by IL-1β and is treatable by biologic agents that block the effects of this cytokine.

## Introduction

It is well known that IL-1β is a constitutive component of the mixture of pro-inflammatory cytokines that are responsible for the inflammation occurring in patients with IBD and, in fact, elevations in IL-1β levels are associated with increased disease severity (1, 2). Nevertheless, in most cases of IBD, there is little evidence that IL-1β plays a dominant or singular role in the inflammatory process and that for the most part, IL-1β acts in conjunction with other pro-inflammatory cytokines such as IL-6 and TNF-α to induce IBD inflammation. This does not mean that the absence of IL-1β does not diminish inflammation in IBD or that pathologic increases in IL-1β secretion cannot itself cause or exacerbate IBD. In the following review, we will address these possibilities with an analysis of the available studies in which IL-1β secretion or signaling is altered in various ways.

## NLRP3 deletion and experimental intestinal inflammation

In the last decade a number of studies have appeared that provide data on the effect of IL-1β deficiency on the development of IBD. These have taken the form of studies of the relation of the NLRP3 inflammasome (a major source of mucosal IL-1β and the inflammasome most subject to activation by multiple aspects of inflammation-associated stress) to intestinal inflammation or studies in which the effect of IL-1β deletion or inactivation on the development of intestinal inflammation in various models has been assessed. One of the first studies exploring the role of NLRP3 in intestinal inflammation were those of Bauer et al. who found that mice lacking NLRP3 (and thus exhibiting decreased IL-1β secretion) were characterized by decreased (but not absent) DSS-colitis and TNBS-colitis compared to control mice (3, 4). Since mice with NLRP3 deficiency in this study also exhibited increased numbers of CD103+ cells (presumably tolerogenic dendritic cells) this protective effect was attributable to increased development of regulatory T cells that suppress the inflammation, but no evidence was presented to support this possibility. Another finding was that the protective effect of NLRP3-deficiency seemed to depend on the gut microbiome, since co-housing the deficient mice with wild type mice for 2 weeks led to normal susceptibility to colitis, i.e., the co-housed NLRP3-deficient mice were no longer protected from colitis by the absence of NLRP3. This observation presaged findings in more recent studies exploring how changes in NLRP3 inflammasome affect the colitogenicity of the gut microbiome that may then have important effects on gut inflammation (5); these are described in greater detail below.

Another study of NLRP3 inflammasome deficiency in which the deficiency led to decreased colitis was based on the finding that a microRNA, miR-223, inhibits NLRP3 generation and is increased in IBD (Crohn's disease) patients, the latter perhaps serving as a mechanism to temper the severity of inflammation in such patients (6, 7). Taking advantage of this finding, Neudecker et al showed that mice lacking miR-223 in hematopoietic cells and thus displaying increased NLRP3 levels in such cells exhibit enhanced DSS-colitis associated with enhanced NLRP3 inflammasome activity (6). The therapeutic potential of this finding was then suggested by studies showing that over-expression of miR-223 via nanoparticle delivery of a synthetic miR-223 inhibits DSS colitis and might similarly inhibit Crohn's inflammation.

At first glance, the above studies showing that NLRP3 inflammasome deficiency results in decreased intestinal inflammation appear to provide strong evidence that a normal level of IL-1β secretion is an important, if not critical component of intestinal inflammation. However, this conclusion is premature because there are numerous additional studies showing that NLRP3 inflammasome deficiency results in increased intestinal inflammation. Before we review these additional studies, however, it is reasonable to point out that the importance of normal levels of IL-1β secretion is an important feature of IBD is in fact buttressed by several studies showing that inhibition of IL-1β function by methods that operate downstream of the NLRP3 inflammasome also results in reduced inflammation. Thus, in the study of miR-233 by Neudecker et al. discussed above, inhibition of IL-1β by a pharmacologic inhibitor (MCC950) or an inhibitor of IL-1βR signaling (anakinra) also attenuated DSS-colitis in the miR-223 deficiency/DSS-colitis model (6). In addition, Seo et al. showed that Ly6C^high^CCR2+ monocytes activated by certain commensal bacteria mediate inflammation in DSS-colitis and that deletion of IL-1β in these monocytes attenuates the colitis (8). Finally, the effect of direct inhibition of IL-1β signaling on intestinal inflammation has been evaluated in mice with chronic granulomatous disease (CGD) due to p40^phox^ deficiency who were administered TNBS per rectum to induce TNBS-colitis (9). It was found that such mice develop more severe TNBS-colitis than similarly treated WT mice, likely as a consequence of the fact that the CGD defect causes increased NLRP3 inflammasome activation. This possibility was supported by the fact that administration of anakinra, an agent that blocks IL-1β signaling, attenuated the inflammation. Thus, in this case, down-stream inhibition of IL-1β blocked NLRP3 inflammasome activity. It should be noted that whereas two patients with CGD and Crohn's-like colitis in this study also responded to anakinra treatment, at least in the short term, blockade of IL-1β or its signaling has not been proven to be an effective treatment in conventional Crohn's disease not associated with CGD.

Returning now to studies of the effect of NLRP3 inflammasome defects on intestinal inflammation we need to consider, as briefly mentioned above, a set of studies which show that such deficiency results in increased intestinal inflammation in various murine models of inflammation. An important initial report in this study set is that of Zaki et al. (5) who showed that mice with NLRP3 deficiency exhibit more severe DSS-colitis than WT mice; in addition, mice lacking ASC and caspase-1 were also subject to increased DSS-colitis suggesting that the latter occurred with deficiency of any of the components of the NLRP3 inflammasome. In bone marrow chimera studies this increased inflammation was traced to decreased expression of NLRP3 in epithelial cells since NLRP3-deficient mice reconstituted with WT bone marrow cells exhibited colitis equivalent to that in NLRP3-deficient mice reconstituted with NLRP3-deficient bone marrow and, conversely, WT mice reconstituted with NLRP3-deficient bone marrow exhibited colitis equivalent to that in wild type mice. In further studies these authors found that mice with deficient ASC or caspase-1, exhibited extensive evidence of decreased epithelial integrity such as increased numbers of TUNEL-positive cells and decreased numbers of proliferating epithelial cells. Since epithelial cells from caspase-1 deficient mice exhibited reduced epithelial cell production of IL-18, these manifestations were attributed to the possibility that NLRP3-deficient mice exhibited reduced epithelial production of IL-18, a cytokine previously shown to help maintain epithelial layer survival in the face of stress (10) and indeed exogenous provision of IL-18 reduced the pro-inflammatory effect of decreased inflammasome function. Finally, Zaki et al. showed that mice with NLRP3 or caspase 1 deficiency, exhibited increased invasion of organisms into the lamina propria and circulation indicative of decreased epithelial barrier function and colitis in these mice was ameliorated by antibiotic treatment (5). Overall, these Zaki et al. studies led to the conclusion that the protective function of the NLRP3 inflammasome in DSS-colitis (implied by increased colitis in mice with NLRP3 deficiency and lack of NLRP3 inflammasome activity) is due to its support of epithelial barrier function via induction of epithelial cell IL-18 secretion. A more recent study by Yao et al., however, calls this conclusion into question: Yao et al., showed that epithelial cells exhibit very little NLRP3 inflammasome activity and the production of IL-18 by these cells results from NLRP3-independent caspase-1 expression; in addition, lamina propria leukocytes produce very little NLRP3-dependent IL-18 (11). Thus, on the basis of these data, the idea that NLRP3-deficiency results in increased colitis because of loss of IL-18-mediated preservation of epithelial barrier function does not hold water. In addition, the fact that administration of exogenous IL-18 reverses the increased inflammation associated with NLRP3-deficiency does not rescue this idea because administration of exogenous IL-18 is not equivalent to IL-18 production by epithelial cells.

Findings parallel to those of Zaki et al. were reported by Dupaul-Chicoine et al. (12) who focused on the effects of caspase-1 deficiency rather than NLRP3-deficiency. These investigators also showed that caspase-1-deficiency led to increased severity of DSS-colitis associated with abnormalities of epithelial cells that was ameliorated by exogenous IL-18. In additional studies, these investigators studied mice that lacked caspase-12, a factor previously shown to bind to caspase-1 and inhibit its function (as well as caspase-1-dependent inflammasomes) (13); these mice were thus the obverse of those with NLRP3 deficiency in that they had increased NLRP3 inflammasome function. As might be expected, the caspase-12-deficient mice were resistant to acute DSS-colitis, presumably due to increased caspase-1 activity and increased production of IL-18. However, caspase-12 deficient mice subjected to sustained DSS treatment exhibited increased DSS-induced inflammation presumably because under these conditions the pro-inflammatory (IL-1β-mediated) effect of the hyperactive NLRP3 inflammasome overcame the epithelial regenerative (IL-18-mediated) effect of increased caspase-1 activity in epithelial cells. This possibility was supported by the finding that mice subjected to chronic DSS-colitis produced by administration of DSS at intervals interspersed with periods of rest were resistant to DSS-colitis, consistent with the idea that during periods of rest the NLRP3 inflammasome is quiescent and the epithelial cell layer can undergo IL-18-mediated repair. Finally, caspase-12 deficient mice with hyper-active NLRP3 inflammasome activity manifested increased azoxymethane (AOM)-induced tumorigenesis when AOM is administered to mice with DSS-colitis. Evidently, the increased survival and proliferation of epithelial cells resulting from a hyperactive inflammasome rendered them more susceptible to neoplastic transformation.

These studies, as well as those of Zaki et al. (5) and Allen et al. (14) (the latter discussed below) insofar as they explored the effect of caspase-1 deficiency as a surrogate for a NLRP3 defect are challenged by the recent discovery that the caspase-1-deficient mice used in these studies were generated using ES cells from the 129 mouse strain that bears a natural mutation in the caspase-11 gene that is not easily separated from a mutated caspase-1 gene by recombination because these gene are too close together; thus the caspase-1-deficient mice were, in reality, carrying a “passenger mutation” and were deficient in both Caspase-1 and Caspase-11 (15). This impacts the interpretation of studies of presumed caspase-1 KO mice because caspase-11 deficiency itself causes immune abnormalities (such as decreased non-canonical NLRP3 inflammasome activation) as well as the development of a colitogenic microflora that leads to increased severity of DSS-colitis not due to caspase-1 deficiency in some studies (16, 17) but not in others (18). Thus, in a study using mice in which the effect of caspase-1 deficiency was assesed in C57BL/6N mice not bearing the natural caspase-11 mutation and in which control mice harbored an equivalent gut microflora, caspase-1 deficiency was shown to cause decreased DSS-colitis and decreased production of epithelial cell IL-18 that was not dependent on changes in gut microflora (18). This result is obviously very different from that of Zaki et al. and Dupaul-Chicoine et al and calls into question that loss of increased IL-18 production explains the more severe colitis in mice with caspase-1 deficiency. It also conflicts with the studies of caspase-12 deficient mice that had decreased DSS-colitis and increased caspase-1 activity. In contrast, it fits nicely with the aforementioned studies of Yao et al. (11), that posited that epithelial caspase-1 secretion was not under NLRP3 inflammasome control. In addition, these new data relating to caspase-1 correlate with studies showing that IL-18 causes increased DSS-colitis when studied under carefully controlled gut microbiota conditions (19).

Two other studies assessing the effect of NLRP3 inflammasome deficiency on experimental colitis have appeared which provide additional information regarding the effect of this deficiency. In one of these studies, Allen et al. showed that mice lacking ASC and caspase-1 manifested increased DSS-colitis severity as well as AOM tumorigenesis (14); this was also seen in mice lacking NLRP3 albeit to a lesser degree. In contrast, mice lacking NLRC4 were not different from WT mice with respect to level of DSS-induced inflammation. Since the NLRC4 inflammasome is activated by flagellin and the type III secretion system complex (T3SS) bound to NAIP5 this indicated that intestinal inflammation induced by DSS is not triggered by pathologic organisms that interact with and activate NLRC4 via flagellin or T3SS. Of interest, in this study reconstitution of mice with NLRP3-deficient hematopoietic cells was also associated with increased DSS-colitis and AOM-induced tumorigenesis. This finding further reduces the likelihood that NLRP3-deficiency results in increased colitis due to lack of IL-18 production in epithelial cells as suggested by Zaki et al.

In another of these studies Hirota et al. (20) showed that DSS-colitis (and in this case TNBS-colitis as well) was more severe in the absence of inflammasome function and IL-1β production. In this work, NLRP3 deficiency was associated with reduced levels of regulatory cytokines, IL-10 and TGF-β in contrast to the study of Bauer et al described above in which NLRP3 deficiency was associated with reduced inflammation and increase numbers of tolerogenic dendritic cells. In addition, the NLRP3 deficiency was accompanied by reduced neutrophil chemotaxis and increased neutrophil apoptosis. Finally, this deficiency was also accompanied by various changes in defensin production and reduced anti-microbial capacity of crypt secretions as well as an altered intestinal microbiome of unknown significance. In this report several of the observed consequences of NLRP3 deficiency, such as the occurrence of decreased neutrophil function, do not correlate with the increased inflammation whereas other consequences do correlate, such as the presence of decreased regulatory cytokine levels. Nevertheless, this report is valuable because it emphasizes that loss of IL-1β secretion has far-ranging down-stream effects that can also influence the severity of inflammation.

The several studies of the effect of NLRP3 inflammasome deficiency on experimental colitis (mainly DSS-colitis) summarized above provide a mixed and therefore confusing picture concerning the role of IL-1β in experimental colitis (and, by extension, in Crohn's disease). We have already discussed some of the problems with the interpretation of the findings in these studies and thus their limitations. At this point, it is appropriate to make several additional comments concerning the assessment of these studies that might help explain their contradictory findings.

First it is important to keep in mind that the NLRP3 inflammasome has several important effects on intestinal immune responses that transcend inflammasome generation of IL-1β. For instance, the inflammasome via its activation of caspase-1 generates a mature form of gasdermin-D, a factor that causes perforations in the cell membrane and mediates a form of cell death called pyroptosis; for this reason, activation of the NLRP3 inflammasome can result in death of effector macrophages/dendritic cells within the site of inflammation (21). Second, another effect of NLRP3 inflammasome already noted above (11), is that NLRP3 up-regulation induces effects on the gut microbiome that increases its capacity to influence regulatory T cells; thus, NLRP3 deficiency may lead to reduced regulatory cell numbers and, as a result, increased mucosal inflammation. As discussed in greater detail below, effects of inflammasomes on the gut microbiome is not limited to upregulation of NLRP3 inflammasome activity, it can also result in down-regulation of inflammasome activity in that it is seen in studies of mice with NLRP3, NLRP6, NLRP12 deficiency (4, 22, 23).

Finally, difficulties in the evaluation of the pro-inflammatory effects of IL-1β in studies of DSS-colitis in NLRP3-deficient mice arise from studies conducted by Bersudsky et al showing that damaged epithelial cells resulting from DSS exposure or exposure to microbiota leads to release of IL-1α, a pro-inflammatory “alarmin” that is not dependent on an inflammasome for its generation. Moreover, these studies aver that IL-1α rather than IL-1β is responsible for tissue injury in DSS-colitis since neutralization of the former but not the latter leads to alleviation of the inflammation and IL-1β secretion supports epithelial cell layer healing and repair (24). These “contrarian” studies require confirmation by other investigators and, if validated, can explain the increase colitis associated with NLRP3 inflammasome dysfunction. However, it is clear from numerous other studies that IL-1β does exert proinflammatory function and thus the anti-inflammatory function of this cytokine in these studies raise new questions about the role of this cytokine during inflammation that will require further study.

## The role of inflammasomes other than NLRP3 in experimental colitis

In that either increased or decreased activity of the NLRP3 inflammasome affects the level of inflammation in experimental colitis it is important to ask if other inflammasomes also affect the severity of DSS-colitis or any other type of experimental colitis. In a study by Allen et al., already discussed above, it was shown that deletion of the NLRC4 inflammasome had no effect on the severity of DSS-colitis, at least in mice harboring commensal organisms as in studies of the NLRP3 inflammasome (14). In another study by Chen et al., the effect of deletion of the NLRP12 inflammasome, an inflammasome previously shown to cause accelerated IL-1β secretion (but no overall increase in IL-1β secretion) and to inhibit NF-κB activation (25, 26) was explored (23). It was shown that NLRP12 deficiency led to increased DSS-colitis associated with a less diverse microbiome characterized by decrease in the concentration of commensal strains that protect from colitis associated with increase in the concentration of commensal strains that induce colitis; thus, this inflammasome was thought to affect the severity of the DSS-colitis by affecting the colitogenicity of the microbiome by an as yet undefined mechanism.

In yet another study (or rather series of studies) of the effect of inflammasomes other than the NLRP3 inflammasome on experimental colitis Elinav and his associates assessed the role of NLRP6 in DSS-colitis (22). NLRP6 is an inflammasome expressed mainly in epithelial cells that results in processing and secretion of IL-18 but not IL-1β; thus the activity of this inflammasome is only indirectly related to activity of inflammasomes resulting in IL-1β secretion (27).

In these studies evidence was provided showing that epithelial cell NLRP6 deficiency results in reduced IL-18 secretion and the appearance of an altered microflora containing increased numbers of bacteria belonging to the Bacteroidetes (Prevotellaceae family) and TM7 phyla; indeed, numerous Prevotella organisms could be identified in colonic crypts of the deficient mice. Similar findings were found in mice with genetic deletions causing ASC-, caspase-1- and IL-18-deficiency, the latter identifying IL-18 secretion as necessary for the prevention of the expansion of Prevotella-like organisms in the mucosal lumen adjacent to epithelial cells. The microflora developing in NLRP6-deficient mice was characterized as “colitogenic” because it was associated with mucosal hyperplasia, an influx of inflammatory cells and more severe DSS-colitis; in addition, it caused a similar colitis when transferred to control mice. In further studies focusing on the mechanisms underlying this colitogenicity, it was shown that the NLRP6 inflammasome is regulated by factors generated by the microflora that act as positive (e.g., taurine) or negative (e.g., spermine) inflammasome regulatory signals. Wild type (normal) microflora elaborate a positive set of signals that leads to NLRP6 activation and the generation of IL-18 as well as IL-18-induction of anti-microbial peptides (AMPs) that continue to maintain a normal (non-colitogenic) microflora. Contrariwise, in NLRP6 deficiency both IL-18 secretion and IL-18-induction of AMP secretion is curtailed and a dysbiotic microflora ensues. The latter generate a negative set of second signals that can suppress normal NLRP6 inflammasome function in a second host to which it has been transferred and thereby exercise colitogenicity (28).

Overall, these findings were notable because they showed, for the first time, that a single genetic abnormality could influence the make-up of the gut microflora and that the latter could, in turn, cause dysbiosis and thus epithelial abnormalities and increased susceptibility to a colitis-inducing environmental agent, in this case, DSS. However, these findings were subsequently challenged by Mamantopoulos et al. who also performed studies of mice with NLRP6 or ASC deletions and found that the deficient mice were not different from control mice with respect to mucosal microflora or response to DSS administration when compared to littermate control mice sharing the same intestinal microflora (29). They thus concluded that the prior studies of NLRP6 were due to “legacy” effects resulting from differential maternal inheritance and/or long term separate housing. On this basis, the effect of NLRP6 mutations would only develop in particular mouse sub-strains and/or microbial environments. In yet additional studies that counter this diminished view of the ability of host genetic factors affecting the NLRP6 inflammasome to influence the microbiome, it was shown in several laboratories that reconstitution of germ-free WT and NLRP6-deficient mice with a complex microflora results in NLRP6-dependent changes on microfloral colitogenicity as originally described. This suggests that phenomenon is broadly applicable to most if not all environments (30–32). In the light of these studies relating to NLRP6 (and IL-18), it is possible that many of the findings reviewed above showing differences between NLRP3-deficient mice and control mice with respect to IL-1β and experimental colitis must be re-examined in studies in which deficient mice are compared with littermate control mice or mice that otherwise bear an identical microbiome.

## Increased production of IL-1β and IBD

Another way to interrogate the role of IL-1β in IBD is to determine the effect of increased production of this cytokine on mucosal homeostasis and the development of intestinal inflammation. As in the case of models in which IL-1β secretion is decreased, models in which IL-1β secretion is increased also involve NLRP3 inflammasome abnormalities, but in this case the abnormality is NLRP3 inflammasome hyperactivity rather than lack thereof.

**Figure 1:**
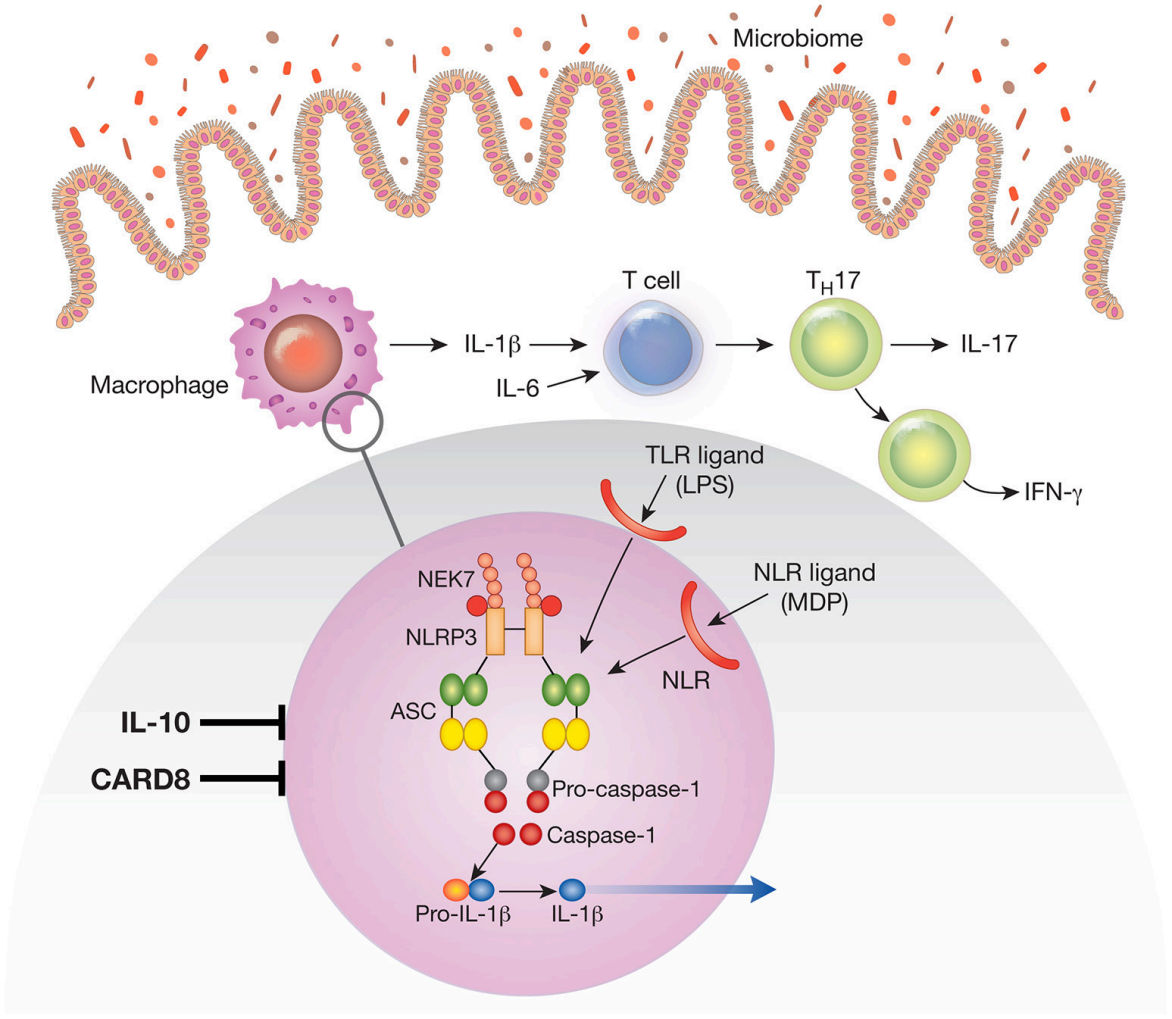
The NLRP3 inflammasome existent in mucosal macrophages and dendritic cells provides the cellular “machinery” for the generation of the mature (cleaved) form of IL-1β secreted by these cells. In this figure the NLRP3 inflammasome is depicted in the blow-up of an area of macrophage cytoplasm present in the small circle. Note that the NLRP3 inflammasome can be activated by various TLR ligands (such as LPS) or NLR ligands (such as the NOD2 ligand, muramyl dipeptide); these are derived from the gut microbiome and act as first signals that induce synthesis of inflammasome components. Not depicted are second signals such as ATP and nigericin that act as second signals that induce inflammasome component assembly and function. Cells containing the NLRP3 inflammasome in the intestinal lamina propria secrete IL-1β and the latter in concert with IL-6 induces na.ve T cells to differentiate into Th17 T cells that secrete IL-17 or downstream T cells that secrete IFN-γ; these are the main cells that create and sustain the inflammation. During an inflammation such as that induced experimentally by exposure to dextran sulfate (DSS-colitis) or by factors causing human inflammatory bowel disease (IBD) IL-1β functions in tandem with other proinflammatory cytokines such as TNF-α and IL-6 to mediate inflammatory changes in the lamina propria. Under these circumstances treatment that counteracts IL-1β pro-inflammatory effects alone has little or no efficacy. However, a different situation obtains when the function of a factor that ordinarily inhibits the NLRP3 inflammasome, such as CARD8 or IL-10 is impaired. Thus, in the presence of mutations or other conditions leading to defective CARD8 function or to defective IL-10 signaling (IL-10 deficiency or IL-10R deficiency), NLRP3 inflammasome function and IL-1β secretion is greatly increased. Under these circumstances IL-1β becomes a more dominant component of the inflammatory process and the latter becomes more susceptible to treatment that counteracts IL-1β effects.

In the discussion above we have already encountered one such model, DSS-colitis in mice with caspase-12 deficiency, i.e., mice lacking an inhibitor of the NLRP3 inflammasome and of caspase-1 and have seen that this abnormality gives rise to increased NLRP3 activity causing less severe acute DSS-colitis and more severe chronic DSS-colitis reflecting the relative effect of NLRP3 and caspase-1 hyperactivity on IL-1β and IL-18, respectively. A less esoteric setting of NLRP3 hyperactivity is colitis occurring in the presence of IL-10 deficiency which is due to the fact that IL-10 has the unique ability among commonly produced cytokines to inhibit the NLRP3 inflammasome and thus to cause hyperactivity of this inflammasome in its absence (33) (see Figure 1). The first study to examine how this IL-10 property could affect colitis was conducted by Zhang et al. (34) who showed that IL-10-deficient mice exhibited increased NLRP3 inflammasome activity and IL-1β production and that inhibition of this activity in colonic explants with IL-1βR antagonist or with a caspase-1 inhibitor decreased both IL-1β and IL-17 secretion in the explant. More importantly, they showed that administration of IL-1βR antagonist to IL-10 KO mice led to an impressive decrease in colitis. In a second and somewhat more complete study of this IL-10 effect conducted some years later, Liu et al. showed that NLRP3 inflammasome activity and IL-1β secretion is increased in IL-10 deficient mice even before the spontaneous onset of colitis and this hyperactivity is observed in both macrophages and epithelial cells (35). Furthermore, inhibition of the NLRP3 inflammasome by administration of glyburide, an agent that inhibits ATP-sensitive potassium K+ channels, led to greatly decreased colitis. Expanding on the Zhang et al study, Liu et al showed that while such treatment directly affected only IL-1β, a host of pro-inflammatory factors were indirectly decreased, indicating that IL-1β has potent down-stream effects. Finally, Liu et al showed that these findings were applicable to Crohn's disease in that colonic explants from patients with this disease also exhibited reduced pro-inflammatory cytokine production when cultured with glyburide.

A further study of the effect of IL-10 deficiency as it relates to the role of IL-1β-mediated inflammation was conducted in both mice and patients with IL-10R deficiency, the latter with early onset IBD (36). In initial studies centered in mice it was shown that RAG1-deficient/IL-10Rβ-deficient mice adoptively transferred IL1-deficient CD4+ T cells manifested less colitis and less pro-inflammatory cytokine production than the same mice transferred WT CD4+ T cells presumably because in the former instance the transferred IL-1r-deficient T cells were not responsive to the excessive IL-1β produced in mice whose NLRP3 inflammasomes was not inhibited by IL-10. In subsequent studies in humans it was shown that human IL-10R-deficient macrophages were stimulated by LPS alone to secrete IL-1β via caspase-8-dependent inflammasome activation, reflecting a hyperactive NLRP3 inflammasome that did not require a second signal such as ATP or nigericin. More importantly, treatment of two patients with IL-10R deficiency and severe IBD unresponsive to conventional therapy exhibited marked improvement with IL-1R antagonist, (anakinra) administration. These data thus fully corroborated earlier findings in mice with IL-10 deficiency treated with NLRP3 inflammasome inhibitors and extended these findings to humans with IL-10R abnormalities.

Yet another experimental model of colitis where IL-10-deficiency facilitates IL-1β proinflammatory effects comes somewhat unexpectantly from studies of TLR5-deficient mice. Thus, as shown by Carvalho et al. while TLR5-deficient mice sometimes develop spontaneous colitis such mice are usually free of colitis unless administered anti-IL-10R (37). Furthermore, this colitis was dependent on IL-1β generation since mice deficient in both TLR5 and IL-1R were protected from such induction of colitis. In addition, the anti-IL10R induced colitis was not mediated by changes in the gut microbiome as the latter was altered in TLR5-deficient mice treated with anti- IL-10R whether or not they developed colitis. The authors considered several possible reasons for this IL-1β effect, including upregulation of several other innate pathways that lead to upregulation of IL-1β secretion. The most interesting of these is activation of the NLRC4 inflammasome by flagellin in mice lacking a potent flagellin-binding receptor. In addition, the authors suggested that TLR5 signaling normally induces IL-1R antagonist expression and its absence thus licenses IL-1β-mediated inflammation.

From the above discussion it becomes apparent that genetic or acquired defects in any of a number of mechanisms involved in the regulation of the NLRP3 inflammasome can conceivably result in abnormalities of the activation of this inflammasome that affect intestinal inflammation. This includes regulation of this inflammasome by such diverse factors as bile acids (38) RNA-dependent Protein Kinase (PKR) (39) and ROS regulation (40).

## Human genetic defects causing excessive IL-1β production and IBD

As mentioned in the Introduction, while IL-1β production in ordinary IBD is increased, this cytokine does not play a decisive (non-redundant) proinflammatory role. This is shown very nicely by the fact, already mentioned, that the ordinary patient with IBD does not respond to treatment with an IL-1β receptor inhibitor such as anakinra. Another fact supporting the concept that IL-1β has limited intestinal proinflammatory capability is that patients with autoinflammatory syndromes [patients with CAPS (cryopyrin-associated periodic syndrome)] that manifest a lowered threshold of NLRP3 activation and produce IL-1β without a second signal only rarely manifest intestinal inflammation. The reason this is the case is not completely clear since we have already discussed the fact that NLRP3 hyperactivity due to lack of IL-10 down-regulation can have proinflammatory consequences in the gut. One possible explanation comes from the recent study by Yao et al. who have shown that mice bearing a NLRP3 mutation that replicates that in patients with CAPS (Nlrp3^R258W^) not only maintain normal homeostasis in the unperturbed gut, they are resistant to colitis induced by DSS administration and neoplasia induced by DSS plus AOM administration (11). Similarly, they are resistant to the development of cell transfer colitis. Investigation of the reason for this increased resistance to induction of experimental colitis disclosed that whereas increased IL-1β secretion due to the mutation led to increased IL-1β secretion(and IL-17 production as well) as expected from the presence of the inflammasome mutation, it was accompanied by decreased production of other pro-inflammatory mediators. This, in turn, was due to increased epithelial cell production of anti-microbial peptides (e.g., Itln1, sPLA2 and Retn1β) as well as an alteration in the gut microbiome that led to the appearance of bacterial species that induced Foxp3+ regulatory cells in the lamina propria. Thus, the ultimate effect of increased NLRP3 inflammasome activity in the lamina propria was protection from induced inflammation due to increased regulatory T cell activity. In view of these findings one might ask why IL-10-deficient mice with secondary increases in NLRP3 deficiency not develop increased regulatory cell activity that prevents colitis. The answer may lie in the fact that IL-10 has been shown to be important for maintenance of Foxp3 expression and regulatory cell suppressor function in colitis (41).

Another genetic disorder leading to increased NLRP3 inflammasome activity in the intestine involves an abnormality in the function of tyrosine phosphatase non-receptor 22 (PTPN22), an enzyme that dephosphorylates NLRP3 at Tyr859 (in mice) and Tyr861 (in humans) (42). Investigation of the function of this phosphatase in relation to the NLRP3 inflammasome showed that maximal activation of this inflammasome occurring during inflammation is facilitated by dephosphorylation of NLRP3 phosphorylation by this phosphatase. Thus, the requirement of the latter's function serves as a check on aberrant inflammasome activity. It follows from this that mice with PTPN22-deficiency manifest decreased NLRP3 inflammasome activity (and decreased IL-1β production) upon exposure to an inflammatory stimulus and therefore exhibit increased DSS-colitis in the same manner as do mice with frank NLRP3-deficiency. In contrast, mice bearing PTPN22 with an autoimmunity-associated gain-of-function mutation causing increased phosphatase function are protected from DSS-colitis. The latter finding is paradoxical since the gain of function mutation leads to increased NLRP3 inflammasome activity. This, however, is compatible with the finding of Yao et al mentioned above in studies of mice with CAPS-like mutation and a hyperactive NLRP3 inflammasome (11); in addition, it is compatible with the observation of Bersudsky et al that IL-1β may preserve epithelial integrity (24). The clinical relevance of these findings relating to PTPN22 phosphatase activity is inherent in the fact that polymorphisms in the genetic region encoding this phosphatase and causing increased phosphatase activity is associated with protection from Crohn's disease although it is associated with increased risk for the development of other types of autoimmune manifestations.

Studies of the effect of increased IL-1β production relative to that normally found in IBD patients has already been discussed above in relation to patients with IL-10R deficiency and patients with CAPS. Another instance in which increased IL-1β secretion has been linked to IBD was reported in Crohn's disease patients bearing a Crohn's disease risk polymorphism in the TPL2 gene that is associated with increased TPL2 expression (43). TPL2 is a signaling protein that is activated (phosphorylated) by pattern recognition receptor (PRR) signaling such as that caused by NOD2 and TLRs and is required for optimal PRR activation of NF-kB and MAPK's that mediate cytokine induction. Thus, increased TPL2 associated with the polymorphism facilitates enhanced pro-inflammatory cytokine secretion. At least in the case of NOD2 the increased TPL2 activation also leads to increased ERK and JNK activation of caspases and “early” activation of the NLRP3 inflammasome. However, while it is clear from these studies that the TPL2 polymorphism results in increased IL-1β secretion it is not clear that this establishes a connection between increased secretion of IL-1β and susceptibility to Crohn's disease since the increased secretion of IL-1β was accompanied by increased secretion of other pro-inflammatory cytokines that could also contribute to increased inflammation in the patients with the polymorphism.

More definite evidence that a genetically determined increase in NLRP3 inflammasome activity and excessive production of IL-1β is the cause of IBD (Crohn's disease) has come from studies of patients with a loss-of-function mutation in CARD8, a protein previously shown to inhibit the NLRP3 inflammasome (44). In these studies, three members of a kindred (the proband, his mother and his aunt), each with Crohn's disease of varying severity, were found to have a mutation in the T60 isoform of CARD8 that exhibited defective inhibition of the NLRP3 inflammasome; consequently, circulating patient monocytes exhibited increased IL-1β and IL-18 secretion upon NLRP3 inflammasome stimulation and the proband had increased serum IL-1β levels. Immunoblot studies of the proband's monocytes revealed that the mutated CARD8 fails to down-regulate the NLRP3 inflammasome because it fails to bind to NLRP3 and thus inhibit its oligomerization as does unmutated CARD8. Interestingly, the mutation was present on only one allele of the CARD8 gene but nevertheless resulted in decreased overall CARD8 function. This was traced to the fact that the mutated CARD8 exerted a dominant negative effect by binding to the unmutated CARD8 and thus inhibiting the latter's ability to bind to NLRP3. Proof that the increased NLRP3 inflammasome-mediated production of IL-1β is the cause of Crohn's disease in these kindred comes from the fact that the proband had severe Crohn's disease that did not respond to anti-TNF-α administration but did respond to agents that block IL-1β activity (initially anakinra and later canakinumab) with the corresponding abdominal symptoms and decrease of fecal calprotectin titers. Patients with CARD8 mutations and excessive IL-1β production leading to Crohn's disease are in principle similar to patients with IL-10 abnormalities since in both cases increased NLRP3 inflammasome hyperactivity is due to lack of an inflammasome inhibitor (see Figure 1). However, the CARD8 mutation provides more definite proof that increased and excessive IL-1β is capable of causing Crohn's disease because in this case the NLRP3 abnormality is not coupled with another abnormality, namely lack of a regulatory factor (IL-10 or IL-10R), that is itself capable of causing gut inflammation.

## Summary

This review of the relation of IL-1β to human IBD establishes that whereas increased or decreased activity of the NLRP3 inflammasome can have varying effects on experimental gut inflammation due to the complexity of effects generated by inflammasome activation or lack thereof, both experimental colitis and colitis in humans with IBD is exacerbated when IL-1β secretion is increased due to various genetic factors. Of particular importance is the discovery that increased NLRP3 inflammasome activity occurring because of loss of CARD8 inhibition of the inflammasome can be a cause of Crohn's disease which responds only to treatment involving IL-1β inhibition. This discovery suggests the need for further studies to determine the frequency of excess IL-1β secretion in Crohn's disease patient populations due to CARD8 mutations or other abnormalities of the NLRP3 inflammasome. In a related vein, this discovery also mandates that Crohn's disease patients not responsive to conventional therapies be investigated to determine if excess IL-β secretion is present and would therefore benefit from administration of an IL-1β blocker.

## Author contributions

LM wrote the manuscript. AK and IF supervised parts of the project. LM and IF drew the figures. WS supervised the project. WS and IF edited the manuscript.

### Conflict of interest statement

The authors declare that the research was conducted in the absence of any commercial or financial relationships that could be construed as a potential conflict of interest.
